# Multisite Field Evaluation of Oil Accumulation and Agronomic Performance in Grain and Sweet Sorghums Engineered for Lipid Hyperaccumulation

**DOI:** 10.1111/pbi.70654

**Published:** 2026-03-25

**Authors:** Yunzhu Chen, Kiyoul Park, Chunhwa Jang, Jung Woo Lee, Mengyuan Wang, Hyojin Kim, Truyen Quach, Ming Guo, Balasaheb V. Sonawane, Sanbon Ch. Gosa, Thomas Elmo Clemente, Andrew D. B. Leakey, Edgar B. Cahoon, DoKyoung Lee

**Affiliations:** ^1^ Department of Crop Sciences University of Illinois at Urbana‐Champaign Urbana Illinois USA; ^2^ Center for Advanced Bioenergy and Bioproducts Innovation University of Illinois at Urbana‐Champaign Urbana Illinois USA; ^3^ Department of Biochemistry University of Nebraska‐Lincoln Lincoln Nebraska USA; ^4^ Center for Plant Science Innovation University of Nebraska‐Lincoln Lincoln Nebraska USA; ^5^ Agroecosystem Sustainability Center, Institute for Sustainability, Energy, and Environment University of Illinois at Urbana‐Champaign Urbana Illinois USA; ^6^ Carl R. Woese Institute for Genomic Biology University of Illinois at Urbana‐Champaign Urbana Illinois USA; ^7^ Department of Agronomy & Horticulture University of Nebraska‐Lincoln Lincoln Nebraska USA; ^8^ Department of Plant Biology University of Illinois at Urbana‐Champaign Urbana Illinois USA

**Keywords:** biomass feedstock, feedstock quality, leaf gas exchange, sorghum, sustainable aviation fuel (SAF), transformation, triacylglycerol (TAG), vegetative oil

## Abstract

Oil sorghum (OS) has been developed by engineering grain (TX430) and sweet (Ramada) genetic backgrounds to accumulate triacylglycerols (TAG) in vegetative tissues as an energy‐dense feedstock for sustainable aviation fuel (SAF) and other biofuels. This study evaluated two TX430 OS lines (TxHO‐2, TxHO‐3) and two Ramada OS lines (RmHO‐1, RmHO‐2) alongside wild‐type (WT) lines in NE and IL over 2 years (2023–2024) to quantify genotype × environment effects on agronomic performance and TAG accumulation. Across four environments, TX430 OS lines showed average TAG concentrations of 15.0 g kg^−1^ in leaves and 12.8 g kg^−1^ in stems, approximately 19‐fold higher than WT. Ramada OS lines accumulated 26.1 g kg^−1^ in leaves and 12.3 g kg^−1^ in stems, approximately 25‐fold and 13‐fold increases over WT, respectively. OS lines in TX430 exhibited an 18% reduction in biomass (8.4 vs. 9.9 Mg ha^−1^ for WT), while Ramada OS lines had similar WT biomass (18.3 vs. 19.9 Mg ha^−1^ for WT). Among TX430 OS lines, TxHO‐2 achieved the highest TAG yield (190 kg ha^−1^), while RmHO‐1 led the Ramada lines (335 kg ha^−1^) due to higher biomass and similar TAG concentration. Enhanced TAG accumulation increased N, P, and K removal in TX430 lines but not in Ramada lines. Structural carbohydrate and ash concentration were unaffected. Overall, results confirm vegetative lipid accumulation as a viable strategy for high‐biomass sorghum, supporting its potential as a dual‐purpose feedstock for SAF. Future work should focus on minimizing biomass yield penalties and improving nutrient use efficiency in oil sorghum systems.

## 1 Introduction

There is a growing demand for additional biofuels from domestic supply chains, including sustainable aviation fuel (SAF) (Ansell 2023; Degirmenci et al. 2023; Holladay et al. 2020; U.S. Department of Energy et al. 2022). SAF is an alternative aviation fuel derived from biomass or waste‐based feedstocks, including plant oils, waste cooking oils, cellulosic biomass, and municipal solid waste (U.S. Department of Energy 2024).

The SAF production process primarily relies on plant‐based feedstocks, but supply is currently very limited relative to demand (U.S. Government Accountability Office 2023). According to the U.S. SAF Grand Challenge's goal, SAF production must reach at least 9.1 million tons (about 3 billion gallons) annually by 2030 and 106 million tons (about 35 billion gallons) of SAF by 2050 (Holladay et al. 2020; U.S. Department of Energy et al. 2022). However, as of 2022, the U.S. produced only about 0.05 million tons (15.8 million gallons) of SAF, far below the target (U.S. Government Accountability Office 2023). Plant‐based oil could be an excellent feedstock for SAF production in addition to lignocellulosic feedstock, including crop residues and purpose‐grown energy crops. However, U.S. domestic production of plant‐based oil was only 14.3 million tons in 2024 (U.S. Department of Agriculture 2024). To meet the rapid growth in US SAF demand, relying exclusively on traditional oil crops is no longer sufficient to meet future supply needs.

Triacylglycerol (TAG) is the primary component of plant oils, which has long aliphatic chains similar to those of hydrocarbon fuels derived from petroleum and serves as one of the key feedstocks for the commercial production of SAF (Durrett et al. 2008; Dyer et al. 2008; Pasa et al. 2022). At present, TAG is mainly derived from palm and oilseed crops, such as African oil palm ( *Elaeis guineensis* ), rapeseed ( *Brassica napus* ), and soybean ( *Glycine max* ) (Harwood et al. 2013). Therefore, developing new TAG feedstock sources has become an urgent priority to enable the large‐scale production of SAF and other high‐value, oil‐based bioproducts (Park et al. 2021, 2025; Wang et al. 2024; Xu and Shanklin 2016).

Vegetative tissues (leaves and stems) of harvested purpose‐grown energy crops can serve as a potential new source of TAG to supplement existing feedstocks (Luo et al. 2022; Parajuli et al. 2020; Park et al. 2025; Vanhercke et al. 2014). Although large‐scale deployment of bioenergy crops raises concerns about land‐use change and competition with food production, these crops can be cultivated on marginal lands where conventional crops are less economically viable, thereby reducing pressure on prime agricultural land (Jang et al. 2024; Jiang et al. 2021). Integrating bioenergy crops into existing systems may also diversify farm income while minimizing competition with food production. C4 grasses, such as high‐biomass sorghum ( *Sorghum bicolor* (L.) Moench), are well‐known for their high photosynthetic efficiency, biomass productivity, and adaptation to dryland climates like those in the U.S. Great Plains (Olson et al. 2012; Rooney et al. 2007). These agronomic traits, combined with adaptability to diverse environments, make sorghum an attractive crop for farmers while supporting the development of sustainable bioenergy feedstock systems. The well‐developed functional genomics toolbox of sorghum also makes it an attractive platform crop for developing vegetative oil production (Bedell et al. 2005; Howe et al. 2006; Mullet et al. 2014; Park et al. 2025; Somerville et al. 2010). From a techno‐economic perspective, the viability of vegetative oil feedstocks depends on achieving sufficient oil yield per unit land area (Cortés‐Peña et al. 2022; Moser 2010). Previous assessments suggest that increasing vegetative TAG accumulation while maintaining high biomass productivity is essential for improving the economic feasibility of biofuel production systems (Cao et al. 2023; Vanhercke et al. 2014; Zale et al. 2016). In the U.S. Midwest, high‐biomass sorghum typically produces approximately 18 Mg ha^−1^ of dry biomass (Gautam et al. 2020; Jang et al. 2024, 2025; Maughan et al. 2012; Schetter et al. 2022). Assuming the TAG concentration is 5% of dry biomass, the potential oil yield could reach about 900 kg ha^−1^ (115 gal per acre), which is approximately 1.3 times greater than the typical oil yield of soybeans (87 gal per acre based on 20% oil of 3036 lbs. per ac) (American Soybean Association 2024). Moreover, after oil extraction, the remaining sorghum biomass, which is abundant in cellulose and hemicellulose, can be further processed into SAF or other biofuels through either thermochemical or biochemical pathways (Billa et al. 1997; Dolciotti et al. 1998; Wang and Tao 2016).

Oil sorghum (OS) was developed with genetic engineering to accumulate energy‐dense TAG in vegetative tissues (Park et al. 2025). Based on a previous study, OS lines accumulated higher TAG in stems and leaf tissues than in wild‐type lines (Park et al. 2025). Triacylglycerol concentration in the stem tissues of OS lines generated from TX430 grain type reached up to 35 g kg^−1^ at the soft dough stage. Meanwhile, the best‐performing OS line, RmHO‐1, derived from Ramada sweet sorghum, exhibited TAG concentrations of 26 g kg^−1^ in leaves and 7 g kg^−1^ in stems (Park et al. 2025). A similar metabolic engineering strategy applied in energycane resulted in a biomass yield penalty in the hyperaccumulating lipid line L13, whereas the low‐TAG line L2 did not show a significant biomass reduction relative to WT (Cao et al. 2023).

To develop an ideal sorghum hybrid for SAF production, which has high biomass production potential with high vegetative TAG accumulation, and the vegetative TAG accumulation strategy should ultimately apply to high‐biomass sorghum hybrids. Before we move to the next step, however, we need to confirm that the transformation of TAG biosynthetic genes should not impact the physiological and agronomic performance of sorghum across a range of environmental conditions in the potential growing region of the central U.S. Therefore, evaluating the effects of transformation of TAG biosynthetic genes in OS lines on physiological and agronomic performance, biomass yield, and feedstock quality across diverse environments is a crucial step in optimizing sorghum genetic modification strategies for high‐biomass sorghum. Our study expanded on the findings of Park et al. (2025) by conducting an evaluation of genotype‐by‐environment (G × E) interactions. The main objective of this field evaluation was to determine the impacts of transformation of TAG biosynthetic genes on changes in crop physiology and agronomic performance of OS derived from two genetic backgrounds (TX430 grain sorghum and Ramada sweet sorghum) across four environments (two locations, Illinois and Nebraska, and 2 years, 2023–2024). To achieve the objective, we compared leaf gas exchange, biomass yield, vegetative TAG concentration and yield, tissue nutrient concentration and removal, and fibre composition (structural carbohydrates and ash concentrations) between OS lines and their respective wild‐type lines.

## 2 Results

### 2.1 Plant Tissue TAG Concentration

**FIGURE 1:**
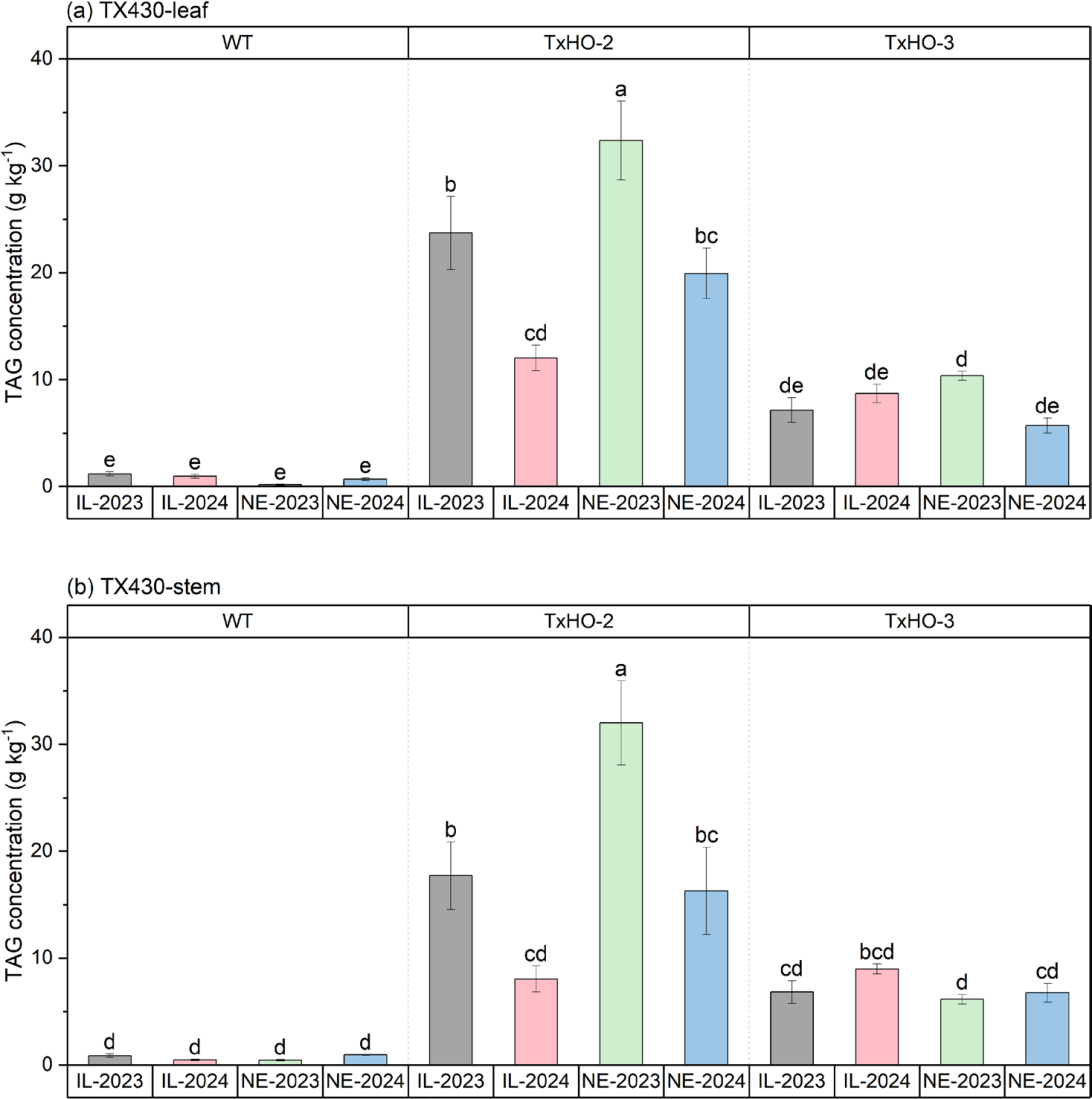
Triacylglycerols (TAG) concentration (g kg^−1^) in (a) leaf and (b) stem tissues under four environmental conditions (IL‐2023, IL‐2024, NE‐2023, and NE‐2024) grouped by genotype of TX430 background (wild‐type (WT) and oil sorghum (OS) lines (TxHO‐2 and TxHO‐3)). The error bars represent the standard error. Lowercase letters indicate mean separation at *α* = 0.05 from highest to lowest value.

In the TX430 background, TxHO‐2 accumulated the greatest TAG concentration in leaves (12–32.4 g kg^−1^) and stems (8.1–32.0 g kg^−1^), although the TxHO‐3 still produced an order of magnitude more TAG than WT (Figure 1a,b). There was significant variation among environments in TAG concentration, which was proportional to absolute TAG concentrations that is, the highest TAG line, TxHO‐2, showed the greatest variation among environments (Table 1). The highest and lowest TAG concentrations were observed in NE‐2023 and IL‐2024, respectively. Additionally, the accumulation patterns in leaf and stem tissues were very similar across genotypes and environments (Figure 1a,b). Similar to TAG concentration, the total fatty acid (TFA) concentration results showed high variation across environments in TxHO‐2's leaf (51.1–83.5 g kg^−1^) and stem (13.9–56.7 g kg^−1^) tissue; nevertheless, the highest TFA was exhibited among the TX430 background (Figure S1).

**TABLE 1:** Analysis of variance (ANOVA) showed the effects of genotype (G), environment (E), and their interaction (G × E) on TAG concentration, dry biomass yield, and TAG yield in TX430 and Ramada backgrounds.

Factor	TAG concentration		Dry biomass	TAG yield
	Leaf	Stem		
TX430				
Genotype (G)	< 0.001	< 0.001	< 0.001	< 0.001
Environment (E)	< 0.001	< 0.001	< 0.001	< 0.001
G × E	< 0.001	< 0.001	0.073	< 0.001
Ramada				
Genotype (G)	< 0.001	< 0.001	0.267	< 0.001
Environment (E)	0.493	0.002	< 0.001	< 0.001
G × E	0.597	< 0.001	0.385	0.005

**FIGURE 2:**
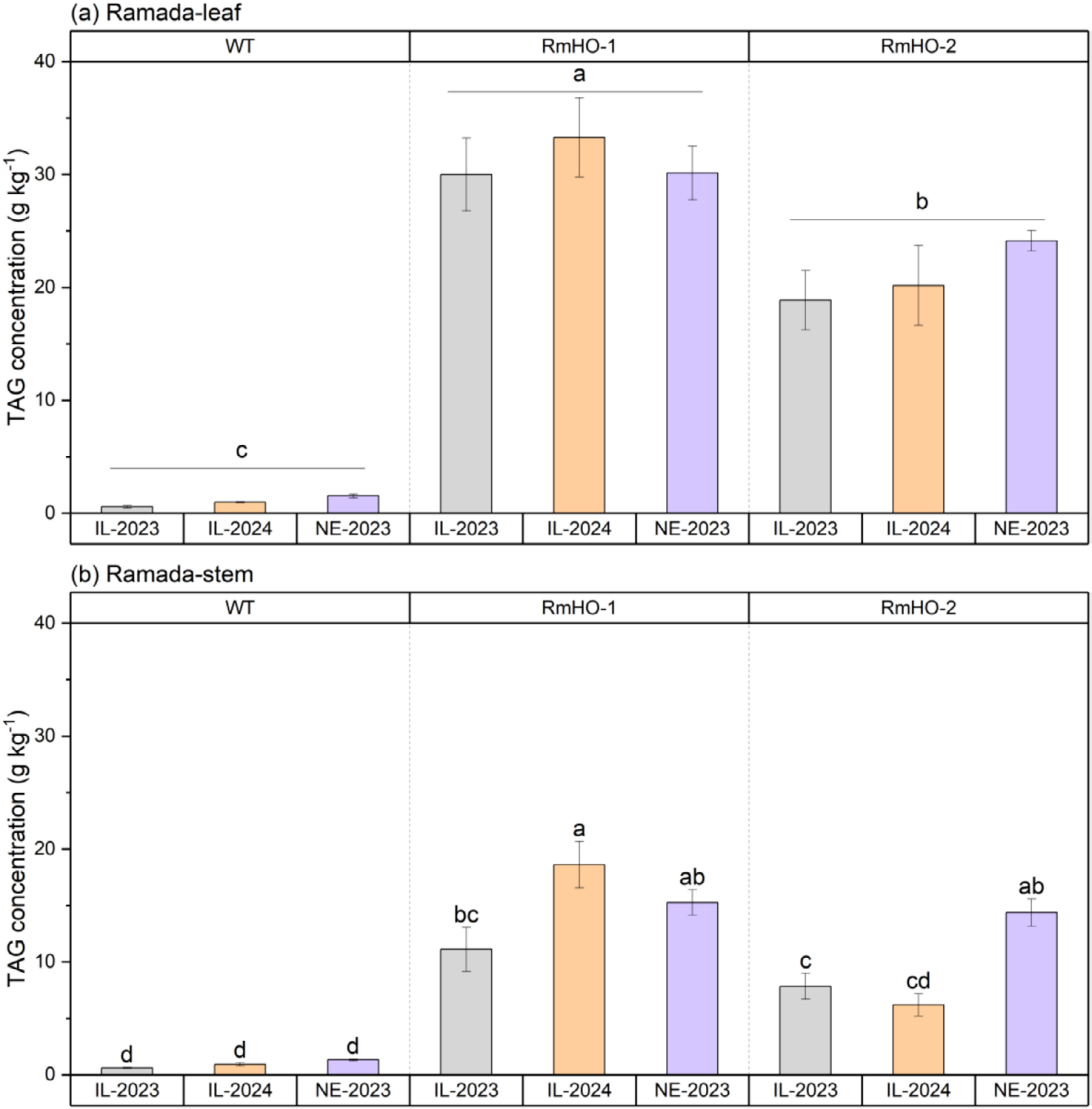
Triacylglycerols (TAG) concentration (g kg^−1^) in (a) leaf and (b) stem tissues under three environmental conditions (IL‐2023, IL‐2024, and NE‐2023) grouped by genotype of Ramada background (wild‐type (WT) and oil sorghum (OS) lines (RmHO‐1 and RmHO‐2)). The error bars represent the standard error. Lowercase letters indicate mean separation at *α* = 0.05 from highest to lowest value.

In the Ramada background, RmHO‐1 accumulated the greatest TAG concentration in leaves (30.0–33.3 g kg^−1^) and stems (18.6–11.1 g kg^−1^), while overall TAG concentrations were approximately twice as high in leaves as in stems (Figure 2a,b). Although RmHO‐2 accumulated less TAG in leaves and stems than RmHO‐1, levels remained significantly higher than WT. The TAG accumulation patterns in leaf tissues were very similar across genotypes and environments (Figure 2a). In contrast, TAG concentrations in stem tissues varied significantly among environments (Figure 2b and Table 1). In particular, RmHO‐1 accumulated higher TAG concentrations in IL‐2023 and IL‐2024 than in NE‐2023, whereas RmHO‐2 exhibited the highest TAG concentration in NE‐2023. RmHO‐1 had a relatively higher TFA concentration in both leaves (52.4–66.1 g kg^−1^) and stems (17.2–26.2 g kg^−1^) across environments than RmHO‐2 (42.7–53.6 g kg^−1^ for leaves and 9.8–19.5 g kg^−1^ for stems) (Figure S2).

### 2.2 Biomass and TAG Yield

**FIGURE 3:**
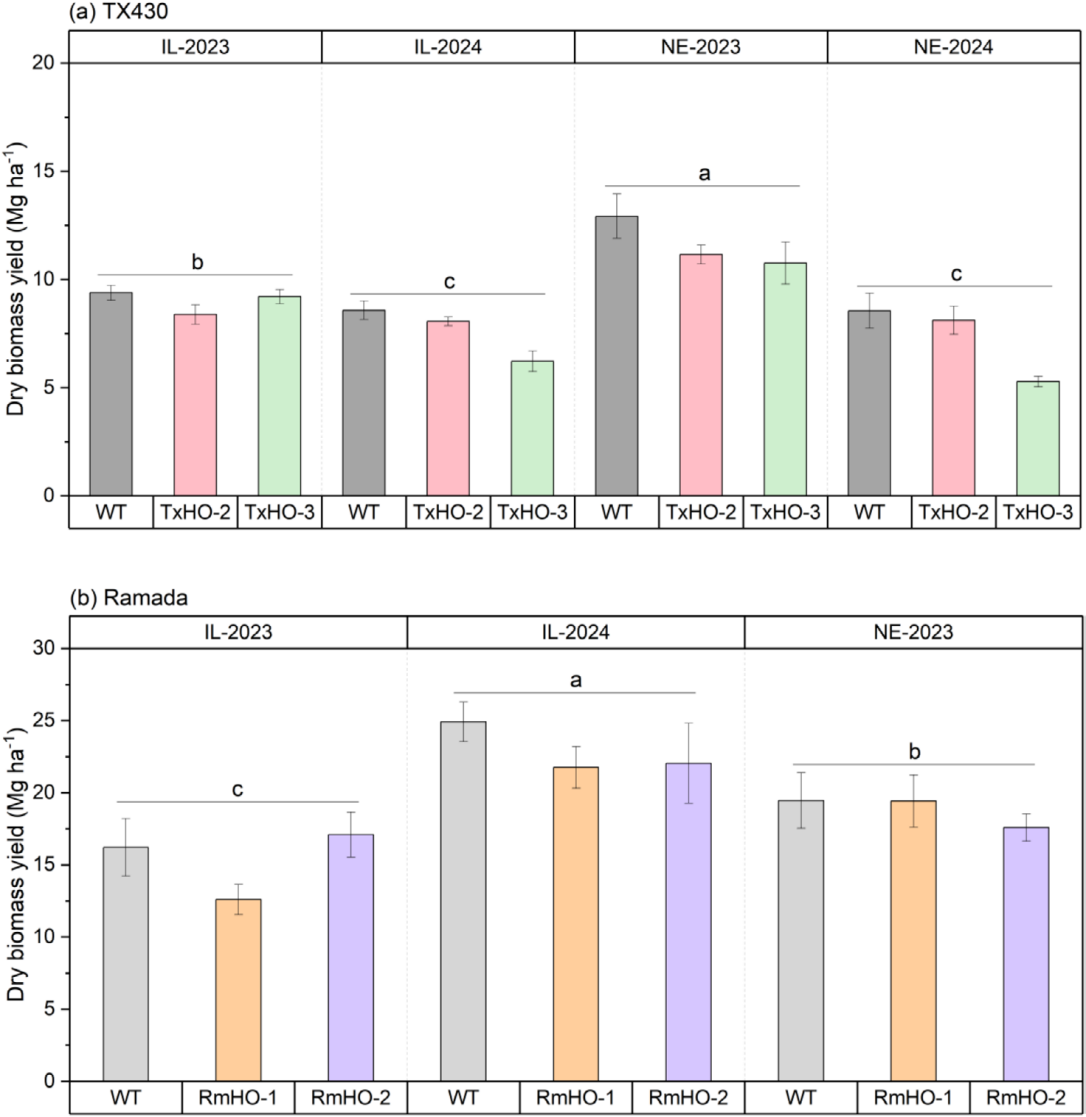
Dry biomass yield (Mg ha^−1^) of wild‐type (WT) and oil sorghum (OS) lines of (a) TX430 and (b) Ramada backgrounds under four environmental conditions (IL‐2023, IL‐2024, NE‐2023, and NE‐2024). OS lines include TxHO‐2 and TxHO‐3 for TX430 and RmHO‐1 and RmHO‐2 for Ramada. The error bars represent the standard error. Lowercase letters indicate mean separation at *α* = 0.05 from highest to lowest value.

In the TX430 background, average biomass production across the 4 site‐years was significantly (*p* < 0.001, Table 1) lower than WT in TxHO‐2 (−10%) and TxHO‐3 (−20%), respectively (Figure 3a). There was no significant variation among environments in the yield drag observed in OS (*p* = 0.073, Table 1). This was despite significant variation among environments in average biomass production across all genotypes, which was driven by higher overall yields in NE‐2023 and lower yields in IL‐2024 and NE‐2024 (Figure 3a).

In the Ramada background, average biomass production across the 4 site‐years was not significantly different between OS and WT lines (*p* = 0.267, Table 1 and Figure 3b). There was no significant genotype × environment interaction (*p* = 0.385), but there was a significant effect of environment driven by greater biomass production in IL‐2024 than NE‐2023 and IL‐2023 (Figure 3b).

**FIGURE 4:**
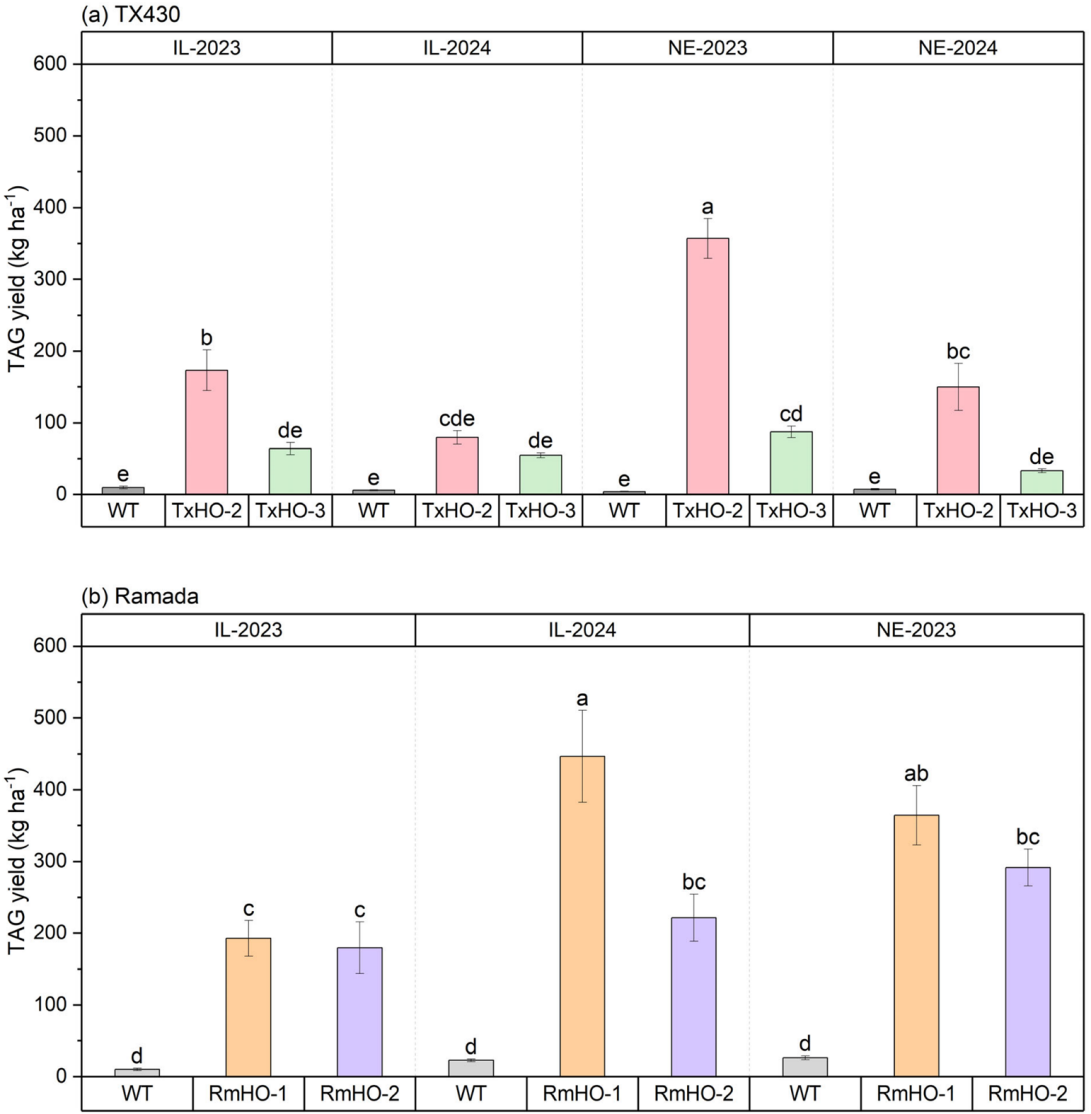
Triacylglycerols (TAG) yield (kg ha^−1^) of wild‐type (WT) and oil sorghum (OS) lines of (a) TX430 and (b) Ramada backgrounds under four environmental conditions (IL‐2023, IL‐2024, NE‐2023, and NE‐2024). OS lines include TxHO‐2 and TxHO‐3 for TX430 and RmHO‐1 and RmHO‐2 for Ramada. The error bars represent the standard error. Lowercase letters indicate mean separation at *α* = 0.05 from highest to lowest value.

The TAG yield was determined by multiplying biomass yield by TAG concentration. For both TX430 and Ramada backgrounds, G by E interactions were significant for the TAG yields (Table 1). The TAG yields of TX430 and Ramada OS lines were higher than their respective WT (Figure 4a,b). For TX430 OS lines, TxHO‐2 yielded the highest TAG yield among all OS lines, ranging from 149.9 to 357.0 kg ha^−1^ across environments (Figure 4a). For Ramada OS lines, the TAG yield of RmHO‐1 was higher than RmHO‐2 in IL‐2024, but there was no difference in IL‐2023 and NE‐2024, ranging from 192.8 to 446.5 kg ha^−1^ (Figure 4b).

### 2.3 Leaf Gas Exchange

**FIGURE 5:**
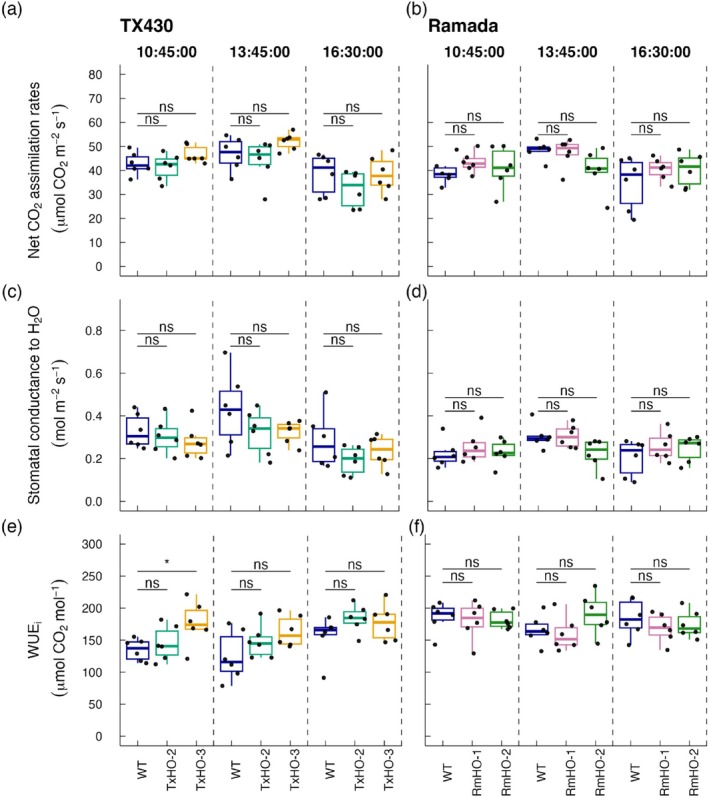
Leaf gas exchange of OS lines and WT lines in TX430 and Ramada background from the IL‐2023 field experiment. Leaf gas exchange variables including net CO_2_ assimilation rates (a and b) stomatal conductance to water vapours (c and d) and intrinsic water use efficiency (WUEi = the ratio of net CO_2_ assimilation rates to stomatal conductance to water; e and f) of OS lines generated in TX430 (a, c, e) and Ramada (b, d, e) background. Measurements were conducted during a sunny day on 68 DAS (22 August 2023) using Li6800 at three time points (10:45, 13:15, and 16:30), separated by vertical dashed lines. Within each box, horizontal lines denote median values; boxes extend from the 25th to the 75th percentile of each genotype distribution of values; vertical extending lines denote adjacent values (i.e., the most extreme values within 1.5 interquartile range of the 25th and 75th percentile of each group); dots denote observations. Outcomes of the unpaired t‐test between WT sorghum line and OS lines are shown above horizontal lines, where ns and * denote *p* is nonsignificant and *p* < 0.05, respectively.

In situ net photosynthetic CO_2_ assimilation rates and stomatal conductance were not significantly different between OS lines and their respective wild‐type lines, in either the TX430 or Ramada genetic backgrounds, at any time of day (Figure 5). Intrinsic water use efficiency (iWUE, net CO_2_ assimilation rate relative to stomatal conductance) was also not significantly different between any OS line and WT, with the exception of TxHO‐3 at mid‐morning, which had significantly greater iWUE than WT (Figure 5e).

### 2.4 Plant Tissue Nutrient Concentration and Removal

In the TX430 background, average tissue N and P concentrations across the 4 site‐years were significantly higher than WT in TxHO‐2 (+8% for N and +4% for P) and TxHO‐3 (+17% for N and 11% for P), respectively (Tables 2 and 3). There was no G × E interaction effect on N and P concentration, while greater environmental effects were observed (*p* ≤ 0.001, Table 2) compared to genotype (*p* = 0.002 for N and *p* = 0.03 for P). In contrast, G × E interactions were observed for tissue K concentration (*p* = 0.002, Table 2), despite the absence of a main genotype effect. The average nutrient removal of N, P, and K in the TX430 background was significantly greater in the WT than in TxHO‐2 (−4% for N, −6% for P, and −12% for K) and TxHO‐3 (−22% for N, −25% for P, and −25% for K), respectively. Across environments, the average removal of N, P, and K was greater in NE than in IL, except for P removal in NE‐2024 (Table 3).

**TABLE 2:** Analysis of variance (ANOVA) showed the effects of genotype (G), environment (E), and their interaction (G × E) on plant tissue nutrient concentrations and removals in TX430 and Ramada backgrounds.

Factor	Plant tissue concentration			Removal		
	N	P	K	N	P	K
TX430						
Genotype (G)	0.002	0.032	0.373	0.002	< 0.001	< 0.001
Environment (E)	< 0.001	0.001	< 0.001	< 0.001	< 0.001	0.013
G × E	0.277	0.189	0.002	0.007	0.006	0.023
Ramada						
Genotype (G)	0.361	0.065	0.584	0.986	0.031	0.256
Environment (E)	< 0.001	< 0.001	< 0.001	< 0.001	< 0.001	< 0.001
G × E	0.977	0.922	0.470	0.889	0.761	0.079

**TABLE 3:** Plant tissue nutrient concentrations and removals in TX430 and Ramada backgrounds for each growing environment in IL and NE during the 2023–2024 growing seasons, shown as mean and standard error in parentheses.

Environment	Genotype	Nutrient concentration (g kg^−1^)			Nutrient removal (kg ha^−1^)		
		N	P	K	N	P	K
TX430							
IL‐2023	WT	12.3 (0.3)	2.7 (0.1)	19.9 (0.6)	115.9 (6.3)	25.5 (1.6)	187.2 (9.4)
	TxHO‐2	13.4 (0.9)	2.9 (0.1)	20.9 (1.0)	113 (11.7)	24.6 (1.7)	177.1 (18.0)
	TxHO‐3	15.2 (0.9)	3.1 (0.2)	23.4 (0.7)	124.3 (12.7)	25.7 (3.8)	192.2 (12.4)
	Mean	13.6 (0.5)	2.9 (0.1)	21.3 (0.6)	117.3 (5.7)	25.2 (1.3)	185.1 (7.7)
IL‐2024	WT	14.6 (1.5)	3.3 (0.3)	22.5 (1.4)	123.8 (11.4)	28.0 (2.2)	193.1 (16.2)
	TxHO‐2	13.1 (1.1)	2.6 (0.2)	19.5 (1.2)	105.1 (7.2)	21.1 (1.5)	158.0 (12.0)
	TxHO‐3	17.3 (1.6)	3.4 (0.3)	21.1 (1.1)	106.7 (11.4)	21.0 (2.4)	131.4 (12.9)
	Mean	14.9 (0.9)	3.1 (0.2)	21.0 (0.8)	112.2 (5.9)	23.5 (1.4)	162.6 (9.8)
NE‐2023	WT	15.6 (0.9)	2.7 (0.1)	16.7 (0.3)	199.8 (16.1)	34.0 (1.9)	215.9 (17.5)
	TxHO‐2	17.5 (0.8)	2.7 (0.2)	20.2 (2.0)	180.3 (20.6)	29.8 (1.9)	222.4 (17.4)
	TxHO‐3	16.9 (1.1)	3.0 (0.3)	18.4 (0.9)	116.6 (12.3)	19.3 (1.8)	183.1 (18.3)
	Mean	16.7 (0.6)	2.8 (0.1)	18.4 (0.8)	165.6 (12.5)	27.7 (1.8)	207.1 (10.5)
NE‐2024	WT	15.6 (0.4)	2.0 (0.1)	27.6 (1.9)	132.1 (10.7)	17.7 (2.5)	237.2 (27.6)
	TxHO‐2	18.2 (0.6)	2.7 (0.2)	21.9 (1.0)	147.7 (13.1)	21.9 (1.6)	180.0 (21.0)
	TxHO‐3	18.6 (0.6)	2.6 (0.1)	22.7 (0.8)	98.0 (4.7)	13.7 (1.0)	119.9 (6.8)
	Mean	17.4 (0.4)	2.5 (0.1)	24.1 (0.9)	125.9 (7.5)	17.8 (1.3)	179.0 (16.1)
Mean		15.7 (0.3)	2.8 (0.1)	21.2 (0.5)	130.7 (4.9)	23.5 (0.8)	183.7 (6.0)
Ramada							
IL‐2023	WT	6.4 (0.7)	1.3 (0.1)	14.5 (1.1)	98.8 (7.6)	20.7 (1.8)	226.1 (14.5)
	RmHO‐1	7.1 (0.5)	1.2 (0.1)	14.9 (1.6)	87.1 (5.4)	14.7 (1.2)	184.9 (17.9)
	RmHO‐2	6.8 (0.7)	1.1 (0.1)	14.0 (1.0)	113.3 (8.8)	18.0 (1.0)	235.5 (20.4)
	Mean	6.7 (0.3)	1.2 (0.1)	14.5 (0.7)	99.7 (4.8)	17.8 (1.0)	215.5 (11.0)
IL‐2024	WT	8.8 (0.6)	1.9 (0.2)	11.9 (0.9)	218.6 (20.2)	47.8 (7.1)	296.1 (28.0)
	RmHO‐1	10.1 (0.8)	1.8 (0.1)	9.9 (0.8)	218 (21.0)	39.1 (2.9)	211.3 (13.2)
	RmHO‐2	9.3 (1.2)	1.6 (0.2)	9.4 (1.0)	216.2 (54.2)	35.3 (7.0)	209.8 (36.9)
	Mean	9.4 (0.5)	1.8 (0.1)	10.4 (0.5)	217.6 (18.3)	40.6 (3.3)	237.3 (17.5)
NE‐2023	WT	5.9 (0.9)	1.0 (0.2)	15.5 (1.6)	118.8 (29.5)	21.4 (5.2)	301.9 (43.7)
	RmHO‐1	6.4 (0.2)	0.8 (0.1)	17.7 (0.9)	122.3 (9.0)	15.3 (1.2)	338.8 (27.9)
	RmHO‐2	5.8 (0.6)	0.8 (0.0)	16.3 (0.9)	104.0 (14.6)	14.5 (1.4)	284.1 (14.5)
	Mean	6.0 (0.3)	0.9 (0.1)	16.5 (0.7)	115.0 (10.9)	17.1 (1.9)	308.3 (17.7)
Mean		7.3 (0.3)	1.3 (0.1)	13.9 (0.5)	141.3 (9.9)	24.6 (1.9)	254.3 (10.5)

In the Ramada background, the environment was the dominant factor affecting tissue N, P, and K concentrations (*p* < 0.001, Table 2). In IL‐2024, the average N and P concentrations across genotypes were 1.5‐ and 1.7‐fold higher, respectively, than in other environments. In contrast, the average K concentration across genotypes in IL‐2024 was 1.5‐fold lower than in other environments (Table 3). There was no difference in tissue N, P, and K concentrations across genotypes within each environment, ranging from 5.8 to 10.1 g kg^−1^, 0.8 to 1.9 g kg^−1^, and 9.4 to 17.7 g kg^−1^, respectively (Table 3). Similar to tissue nutrient concentration, the environment mainly influenced nutrient removal in the Ramada background (Table 2). N and K removal by WT did not differ significantly from OS lines. The magnitude of nutrient removal changes varied by environment, with IL‐2024 showing remarkably high N and P removal compared with the other environments (Table 3). The highest K removal was observed in NE‐2023, consistent with the highest tissue K concentration (Table 3).

### 2.5 Fibre Composition

In the TX430 background, cellulose and lignin concentrations were mainly affected by the environment, while hemicellulose and ash concentrations were affected by the G × E interaction (Table 4). In IL, the highest average cellulose and lignin concentrations across genotypes were 307.5 g kg^−1^ in IL‐2023 and 47.1 g kg^−1^ in IL‐2024, respectively (Figure S3a,c). The lowest concentrations of cellulose and lignin were observed in NE‐2023 with 250.4 and 34.3 g kg^−1^, respectively. In IL, hemicellulose (245.5–275.2 g kg^−1^ for TX430) and ash (81.5–116.3 g kg^−1^) concentrations showed similar within‐genotype patterns, with high or low levels in the same varieties, but the variety rankings changed between years. In NE, only the lowest hemicellulose (252.8–303.0 g kg^−1^) and ash concentrations (83.3–122.2 g kg^−1^) followed similar patterns within genotype, and rankings were not consistent across years (Figure S3b,d). Ash concentrations of TX430 WT in both IL‐2024 and NE‐2024 were approximately 8% and 15% higher than those of OS lines in IL‐2023 and NE‐2023 (Figure S1d).

**TABLE 4:** Analysis of variance (ANOVA) showed the effects of genotype (G), environment (E), and their interaction (G × E) on fibre composition, including cellulose, hemicellulose, lignin, and ash concentration in TX430 and Ramada backgrounds.

Factor	Cellulose	Hemicellulose	Lignin	Ash
TX430				
Genotype (G)	0.099	0.585	0.968	0.342
Environment (E)	< 0.001	< 0.001	< 0.001	< 0.001
G × E	0.841	0.005	0.143	0.001
Ramada				
Genotype (G)	0.977	0.396	0.958	0.290
Environment (E)	< 0.001	0.852	0.028	< 0.001
G × E	0.953	0.872	0.590	0.427

In the Ramada background, fibre composition was mainly affected by environmental factors, except for hemicellulose (Table 4). In general, there was no difference in fibre composition between Ramada WT and its OS lines within the given environment (Figure S4). In IL, average cellulose concentrations across genotypes decreased from 286.6 g kg^−1^ in 2023 to 259.1 g kg^−1^ in 2024, whereas average ash concentrations increased from 47.9 g kg^−1^ in 2023 to 68.6 g kg^−1^ in 2024.

## 3 Discussion

This study advances understanding of the physiological and agronomic performance of TAG‐accumulating oil sorghum (OS) by evaluating genotype × environment (G × E) interactions across four sites and years. Field‐grown OS lines derived from TX430 and Ramada backgrounds accumulated substantially higher TAG in vegetative tissues than their respective WT at the soft‐dough stage, with 19.8‐ and 25.4‐fold increases in leaves and 18.8‐ and 12.9‐fold increases in stems, respectively. Among TX430 OS lines, TxHO‐2 exhibited the highest TAG concentrations in both leaves and stems, consistent with prior greenhouse and field evaluations (Park et al. 2025). TxHO‐2 accumulated up to 32.4 g kg^−1^ TAG in leaves and 32.0 g kg^−1^ in stems on a dry mass basis, similar to concentrations reported previously for this genotype in NE field trials (35.0 g kg^−1^; Park et al. 2025). TAG accumulation in TxHO‐2 was highly environment‐dependent, ranging from 12.0 to 32.4 g kg^−1^ in leaves and from 8.1 to 32.0 g kg^−1^ in stems, whereas biomass yield varied over a narrower range (8.1–11.2 Mg ha^−1^), indicating that vegetative TAG accumulation is more sensitive to environmental variation than bulk biomass production (Nam et al. 2022). In contrast, TAG concentrations in Ramada OS lines were relatively stable across environments; in the highest TAG line, RmHO‐1, leaf TAG ranged from 30.0 to 33.3 g kg^−1^ and stem TAG from 11.1 to 18.6 g kg^−1^. The reduced stem TAG in IL‐2023 is consistent with an early harvest before the soft‐dough stage, imposed by the risk of early frost, as supported by lower growing degree‐days at harvest over the growing season (1261 in 2023 vs. 1456 in 2024; Table S1). Previous work has shown that vegetative TAG peaks before flowering in leaves and at the soft‐dough stage in stems (Park et al. 2025); thus, premature harvest reduces TAG accumulation, whereas delayed harvest increases the risk of post‐maturation TAG degradation (Park et al. 2025). These results highlight the importance of matching harvest timing to the TAG accumulation trajectory to maximise total fatty acid capture in biomass.

Sorghum TAG yield is a function of TAG concentration in vegetative tissues and biomass yield. The transformation of TAG biosynthetic genes translated into large gains in TAG yield at the whole‐plant level. OS lines achieved 18.7‐fold (TX430) and 14.5‐fold (Ramada) increases in TAG yield relative to WT, reflecting simultaneous increases in TAG concentration while maintaining biomass yield. Within TX430, TxHO‐2 exhibited TAG yields ranging from 79.5 to 357.0 kg ha^−1^ across environments, whereas in Ramada, RmHO‐1 achieved 192.8 to 446.5 kg ha^−1^. RmHO‐1 not only accumulated higher TAG concentrations (23.1 vs. 20.3 g kg^−1^) but also produced higher mean TAG yield (334.6 vs. 189.8 kg ha^−1^) than TxHO‐2. Together, the biomass yield and photosynthesis data collected from multisite field conditions indicated minimal yield drag attributable to vegetative TAG accumulation in the tested genetic backgrounds. Across locations (IL and NE) and years (2023–2024), average dry biomass yields were 8.9 Mg ha^−1^ for TX430 backgrounds and 19.0 Mg ha^−1^ for Ramada backgrounds, with strong environmental effects on both genetic backgrounds. OS lines produced biomass yields comparable to their WT parents within each environment. In Ramada backgrounds, no detectable biomass yield penalty was observed with TAG biosynthetic gene transformation. In particular, TxHO‐2 did not impose a detectable biomass yield penalty, while TxHO‐3 showed yield reduction. Similar trade‐offs between lipid accumulation and biomass production have been reported in other metabolically engineered C4 bioenergy crops. For example, engineered energycane lines with very high TAG accumulation exhibited substantial biomass penalties, highlighting the importance of balancing lipid biosynthesis with plant growth processes (Cao et al. 2023). In our study, TxHO‐2 maintained stable biomass production (average 8.9 Mg ha^−1^) across four field environments, corroborating earlier reports of unchanged yield potential in TAG‐engineered TX430 (Park et al. 2025). TxHO‐3 exhibited environment‐specific physiological constraints (Figure S5), showing developed twisted whorls during early vegetative stages (V4‐V5; corresponding to the fourth and fifth fully expanded leaves), and this abnormal morphology became more severe in IL‐2024 under increased growing‐season precipitation. Twisted leaves can retain water in the whorl, restrict stomatal function, limit transpiration, and reduce CO_2_ diffusion, thereby depressing photosynthesis and biomass accumulation (Chaves et al. 2002; Das et al. 2015; Gerik et al. 2003; Ishibashi and Terashima 1995; Pandey and Nagar 2003). In Ramada backgrounds, patterns of dry biomass yield between OS lines and WT indicated that TAG pathway engineering did not adversely affect growth or development. The 1.5‐fold lower biomass yield of Ramada in IL‐2023 relative to IL‐2024 likely reflects delayed planting, lower precipitation, and early frost before peak biomass, consistent with reports that sorghum biomass and vegetative TAG accumulation peak near the soft‐dough stage and are strongly influenced by phenology and photoperiod sensitivity (Olson et al. 2012; Park et al. 2025). Leaf gas exchange measurements conducted at IL‐2023 showed similar CO_2_ assimilation rates and stomatal conductance between OS lines and their corresponding WT in both TX430 grain and Ramada sweet sorghum backgrounds. These results are consistent with previous observations in TAG‐accumulating sorghum (Park et al. 2025) and suggest that the introduction of TAG biosynthetic genes did not impair photosynthetic carbon metabolism in OS, in contrast to reports of altered gas exchange in TAG‐engineered C_4_ sugarcane (Parajuli et al. 2020). Although the present study focused on field‐scale evaluation of vegetative TAG accumulation and agronomic performance, the efficiency of lipid extraction and downstream conversion is also an important factor determining the practical viability of lipid‐based biofuel feedstocks. Recent work by Maitra et al. (2024) highlighted that processing efficiency, including lipid extraction and conversion processes, can substantially influence the amount of recoverable fuel from lipid‐rich biomass. Their findings emphasise that evaluating both biomass composition and downstream processing efficiency is necessary to accurately estimate the biofuel potential of engineered feedstocks. While extraction efficiency was not measured in the present study, our results provide important baseline data on TAG accumulation and biomass productivity across multiple field environments. Integrating extraction efficiency and conversion analyses in future studies will therefore be important for estimating the recoverable oil yield and overall techno‐economic feasibility of oil sorghum as a sustainable aviation fuel feedstock.

For commercial deployment of oil sorghum, nutrient use efficiency and fibre composition are critical alongside biomass yield and lipid accumulation. In Ramada backgrounds, genotype effects on biomass yield, tissue macronutrient concentrations, and nutrient removal were negligible, whereas environmental effects were significant, in agreement with multi‐environment variety evaluation studies (Hoffmann 2012). Nevertheless, prior work has reported substantial genotype‐ and environment‐driven variation in tissue nutrient concentration, nutrient removal, and cell wall composition in (Jang et al. 2024; Schetter et al. 2022), suggesting that future breeding and agronomy studies should explicitly consider these interactions. Nutrient removal patterns observed here align with previous field studies in grain and sweet sorghum that documented large variability in nutrient export driven by site‐specific soil and management conditions (Adams et al. 2015; Powell and Hons 1992; Propheter and Staggenborg 2010; Singh et al. 2012). In the current study, the uniform N rate of 89.7 kg N ha^−1^ applied across all sites was chosen to standardise comparisons but may not represent the agronomic optimum for maximizing TAG accumulation and biomass yield in each environment. Consequently, further research is needed to optimise environment‐ and genotype‐specific field management (M) strategies, including soil fertility management for biomass yield and TAG accumulation. Additionally, the current G × E field evaluation showed that TAG pathway engineering had minimal effects on plant fibre composition across the four environments tested. Feedstock quality in lignocellulosic crops is largely determined by structural carbohydrate (cellulose, hemicellulose, lignin) and ash concentrations (Cotton et al. 2013; Murray et al. 2008). The present study showed that TAG biosynthetic transformation did not significantly alter structural carbohydrate or ash concentrations in TX430 or Ramada backgrounds. TX430 biomass contained higher concentrations of cellulose, hemicellulose, lignin, and ash than Ramada, whereas Ramada produced greater total biomass with lower structural carbohydrate content, consistent with previous reports comparing grain and sweet sorghum types (Dolciotti et al. 1998; Richie and McBee 1991). Because TAG accumulation did not compromise lignocellulosic quality, OS biomass remains suitable for downstream conversion to biofuels or biomaterials after oil extraction, enabling coproduct streams that integrate bio‐oil and lignocellulosic bioenergy. This trait combination will be particularly important when the TAG pathway is introgressed into high‐biomass sorghum backgrounds to simultaneously increase vegetative TAG yield and lignocellulosic biomass supply.

Although TAG yields from first‐generation OS lines remain lower than those of dedicated oilseed crops on a per‐hectare basis, they are notable for an annual C_4_ grass that also produces substantial lignocellulosic biomass. Previous studies showed that TAG accumulation in sorghum leaves can reach approximately 3%–8% of dry biomass when lipid biosynthesis pathways are strongly up‐regulated (Vanhercke et al. 2019). The TAG concentrations observed in this field study fall within the lower range of these values, indicating stable vegetative oil accumulation under multi‐environment field conditions. Similar approaches in other crops have reported varying TAG levels, including ~0.9% in engineered sugarcane stems (Zale et al. 2016), up to 4.4% in oilcane leaves under field conditions (Kannan et al. 2022), and ~16% in tobacco leaves under controlled conditions (Vanhercke et al. 2014). Compared with these systems, the OS lines in this study accumulated moderate TAG levels while retaining high biomass productivity. Consequently, OS could complement conventional oilseed crops by supplying both oil and lignocellulosic biomass from the same production system. However, one important agronomic trait that also needs to be addressed for commercial deployment is lodging resistance. During the field evaluation, we experienced lodging issues with the Ramada background. A critical limitation of the Ramada background is its tall stature and soft stems, which increase susceptibility to lodging under high wind or storm conditions (Esechie et al. 1977; Fedenko et al. 2015; Godoy and Tesso 2013; Gomez et al. 2017; Monk et al. 1984; Worley et al. 1991). Lodging was observed in Ramada OS plots at both IL and NE in 2023, and in NE‐2024, severe storms caused extensive stalk damage and lodging that prevented mechanical harvest (Figures S6 and S7), while TX430 remained largely unaffected. Lodging not only hampers mechanical harvesting but also causes substantial biomass losses; for example, machine‐harvested lodged sweet sorghum can suffer up to 40% yield reduction relative to hand harvest (Fedenko et al. 2015; Gomez et al. 2017; Turhollow et al. 2010). These observations underscore the value of transferring the TAG biosynthetic trait into high‐biomass, strong‐stem sorghum ideotypes with improved standability.

Overall, this multi‐environment field study confirms that engineering TAG biosynthesis in vegetative tissues can substantially increase TAG concentration and yield in sorghum without imposing detectable penalties on photosynthetic performance, biomass yield, nutrient uptake, or lignocellulosic composition. TxHO‐2 (TX430 background) and RmHO‐1 (Ramada background) emerged as lead OS lines combining high TAG accumulation with stable agronomic performance, thereby providing strong proof‐of‐concept for vegetative oil production in a major C_4_ grass. The G × E analysis further demonstrates that both vegetative TAG accumulation and biomass yield should be optimised concurrently to maximise TAG yield per unit land area. These findings support the strategic deployment of TAG biosynthetic pathways into high‐biomass sorghum ideotypes with improved standability and stress resilience, leveraging sorghum's inherent adaptability and yield (Olson et al. 2012; Rooney et al. 2007). Given the high vegetative oil content and retained lignocellulosic value, OS represents a promising dual‐purpose feedstock for large‐scale production of lignocellulosic biomass and renewable lipids suitable for SAF and other advanced biofuels.

## 4 Experimental Procedures

### 4.1 Plant Materials

All OS lines used in the field trials were developed through an agrobacterium‐mediated transformation using two binary vectors (pPTN1569 and pPTN1586) at the University of Nebraska‐Lincoln. The pPTN1569 construct contains the SbWRI1, CpuDGAT1, SiOle, CvLPAT, and CvFatB1 transgenes, while the pPTN1586 construct includes SbWRI1, CpuDGAT1, SiOle, CpuLPAT, and Thio14 transgenes (Park et al. 2025). In this study, we used four OS lines: two lines derived from grain sorghum (TX430), including TxHO‐2 and TxHO‐3, and two lines derived from sweet sorghum (Ramada), including RmHO‐1 and RmHO‐2. The variety names were adopted from Park et al. (2025). TX430 and Ramada sorghum inbred lines have been extensively used for transformation of TAG biosynthetic genes, serving as models for testing transformation techniques (Ali et al. 2008; Freeman 1974; Miller 1984; Murray et al. 2009). TxHO‐2 was transformed with the pPTN1569 construct, while TxHO‐3 was transformed with the pPTN1586 construct. RmHO‐1 and RmHO‐2 were transformed with the pPTN1586 construct. Wild‐type lines (WT) in TX430 and Ramada were used to compare with their OS lines. All seeds were obtained from the University of Nebraska‐Lincoln, and the 2023 and 2024 OS lines were classified as T4 and T5 generations, respectively.

### 4.2 Research Sites and Experiment Design

The field evaluation study was conducted in 2023 and 2024 at two locations: the University of Illinois Fruit Farm in Urbana, IL (IL, 40°04′ N, 88°12′ W), and the Eastern Nebraska Research, Extension and Education Center (ENREEC) in Mead, NE (NE, 41°08′ N, 96°26′ W). The experiment followed a randomised complete block design (RCBD) with six replications for each line. The soils at the IL site consist of about 93% Wyanet silt loam (Fine‐loamy, mixed, active, mesic Mollic Hapludalfs), 5% Drummer silty clay loam (Fine‐silty, mixed, superactive, mesic Typic Endoaquolls). At the site in NE, the soils are 92% Yutan silty clay loam (Fine‐silty, mixed, superactive, mesic Mollic Hapludalfs) and 8% Tomek silt loam (Fine, smectitic, mesic Pachic Argiudolls). Each plot consisted of four rows with 0.7 m between the rows (3.05 × 3.05 m). The individual plots were planted with pre‐packed seeds using a 4‐row plot planter, with a seeding rate of 344 320 seeds ha^−1^. Nitrogen fertiliser was applied as granular urea at a rate of 89.7 kg N ha^−1^ at planting time. Herbicide applications varied by location. In IL, a mix of 2.2 kg a.i. ha^−1^ Atrazine and 1.4 kg a.i. ha^−1^ S‐Metolachlor was applied for weed control, while 1.6 kg a.i. ha^−1^ S‐metolacholor was used in NE. To initiate the field experiment, a permit was obtained from the U.S. Department of Agriculture Animal and Plant Health Inspection Service (APHIS) (permit numbers: 124‐2X9SAEX‐A1 in 2023 and 124‐4HUU561 in 2024). All inflorescences were covered with a head bag at the boot stage to prevent pollen‐mediated gene flow.

### 4.3 Environmental Conditions

The monthly precipitation and average temperature data were obtained from the National Oceanic and Atmospheric Administration (NOAA) at Champaign‐Urbana Willard Airport Station, IL, and Ithaca Station, NE. In IL, the total precipitation was 467 mm during the 2023 growing season, approximately 19% lower than the 30‐year average (574 mm), mainly due to low rainfall in May to July (Figure S8a). The 2024 growing season had approximately 12% higher precipitation than the 30‐year average, with above‐average monthly totals in May, July, and August. In NE, the total precipitation was 464 mm during the 2023 growing season, approximately 15% lower than the 30‐year average (544 mm), with severely low rainfall in May (6 mm) and low rainfall in August to October (Figure S8b). Similar to IL, the 2024 growing season in NE had approximately 11% higher precipitation than the 30‐year average, with above‐average monthly totals in May, July, and August. The average temperatures during the 2023 to 2024 growing seasons (May to October) in both IL and NE were consistent with the 30‐year averages, showing minimal inter‐annual variation and limited fluctuations throughout the season (Figure S8c,d). Before planting in 2023 and 2024, soil samples were collected at two experimental sites in IL and NE at the 0–15 cm and 15–30 cm layers, and the basic physical and chemical properties of the soil were analysed. Soils at the IL and NE locations were representative of typical Midwestern croplands, with pH ranging from 6.3 to 6.8 and moderate organic matter content of 3.5% to 4.4% (Table S2). Field evaluations were conducted over two years in different portions of the same field at each location, ensuring consistent soil conditions across years.

### 4.4 Leaf Photosynthetic Gas Exchange

During the 2023 IL field experiment, measurements of in situ photosynthetic leaf gas exchange were conducted on the youngest fully expanded leaf at three time points (10:45, 13:15, and 16:30) on a sunny day 68 DAS (22 August 2023) using a portable instrument (Li6800, LI‐COR, Lincoln, NE) and well‐established protocols (Gray et al. 2016; Markelz et al. 2011). The photosynthetic photon flux density, humidity, carbon dioxide concentration, and temperature experienced by upper canopy leaves were mimicked inside the gas exchange chamber during data collection. Two leaves were measured in each sub‐plot at each timepoint. Two gas exchange systems were used simultaneously in different blocks of the experiment to ensure timely completion of data collection.

### 4.5 Agronomic Data Collection

The date that the crop developed to the boot stage was recorded when more than 50% of the plants in a plot had reached the booting stage. The target growth stage for biomass harvest was when the plant reached the soft dough stage, with no milk coming out when the grain was crushed. This stage corresponds to the highest oil concentration in the stem, as observed in previous studies (Park et al. 2025). Two harvest timings were applied, as TX430 lines reached the soft dough stage earlier than Ramada, approximately 110 DAS in TX430 and 130 DAS in Ramada (Table S3). However, in 2023 IL, Ramada encountered early frost at 116 DAS and was harvested before reaching the targeted growth stage, as tissue lipids could be degraded after frost (Table S3) (Park et al. 2025). The biomass harvest method in 2023 and 2024 differed depending on the study sites. In both years in IL, the entire two middle rows were harvested using a sickle bar mower. In NE, biomass was harvested from a 1‐m section within the middle two rows using a rice knife in 2023, whereas, in 2024, biomass was harvested the same way as at the IL site. After harvesting, all bagged grain heads were collected and devitalized. The fresh biomass weight for each plot was recorded in the field. In 2024 NE, Ramada plots were destroyed due to a severe windstorm; consequently, biomass harvest was not carried out. A subset of plants from each plot was randomly collected at harvest and oven‐dried at 60°C for 72 h to obtain the moisture content, which was then used to calculate the dry biomass yield. The dried samples were ground through a 2 mm screen for chemical compositional analysis.

### 4.6 Plant Tissue Sampling

For TAG concentration analysis, leaf and stem tissue samples were collected from three randomly selected plants in the middle rows of each plot on the day of harvest. For TX430, entire leaf samples were collected from the 6th nodal leaves, and 2 cm stem samples were taken from the internode between the 6th and 7th nodes. For Ramada, entire leaf samples were collected from the 5th nodal leaves, and 2 cm stem samples were taken from the internode between the 5th and 6th nodes, counting from the bottom of the plant. The samples were immediately frozen in liquid nitrogen and then lyophilized using a freeze‐dryer (FreeZone 2.5, Labconco) for 48 h. After lyophilization, the samples were analysed immediately or stored at −80°C until further analysis.

### 4.7 Lipid Analysis

Freeze‐dried samples were shipped to the University of Nebraska‐Lincoln and analysed for TAG concentration. The detailed analysis methods were described by Park et al. (2025). TAG yield was calculated by multiplying the biomass yield by the TAG concentration.

### 4.8 Plant Tissue Nutrient and Fibre Composition Analysis

Plant tissue nitrogen (N) concentration was determined by dry combustion using the LECOFP‐528 N/Protein Determinator (Leco Inc., St. Joseph, MI). Plant tissue phosphorus (P) and Potassium (K) concentrations were measured using the PerkinElmer Optima 8300 inductively coupled plasma (ICP) spectroscopy (PerkinElmer Inc., Waltham, MA). Nutrient removal by sorghum biomass was calculated by multiplying biomass yield by the corresponding tissue nutrient concentration. Fibre composition analyses followed the ANKOM procedure (Ankom Technology Corp., Fairport, NY). The neutral detergent fibre (NDF) and acid detergent fibre (ADF) analyses were performed sequentially by using the Ankom^200^ Fiber Analyzer equipment and #F57 filter bags. Acid detergent lignin (ADL) was determined using the Daisy II Incubator (Ankom Technology Corp., Fairport, NY) and the 08/05 Ankom protocol. The fibre concentrations were calculated using the following formulas: Hemicellulose was determined as the difference between NDF and ADF; Cellulose was determined as the difference between ADF and ADL; and Lignin was represented by the acid detergent lignin (ADL) value. Ash concentration was measured through loss‐on‐ignition using a laboratory muffle furnace (Harris and Marshall 2017).

### 4.9 Statistical Analysis

All data processing and statistical analysis were performed with the R statistical computing software (version 4.4.1; R Core Team 2024). A linear mixed model (LMM) was applied to determine the genotype (TX430: TxHO‐2 and TxHO‐3; Ramada: RmHO‐1 and RmHO‐2) effect within each genetic background (TX430 and Ramada) across two locations and 2 years (IL‐2023, IL‐2024, NE‐2023, and NE‐2024). The model was set with the genotype and location‐year as fixed factors, while block was treated as a random factor. A Tukey's honestly significant difference (HSD) test was used for posthoc analysis to separate means among OS lines with a significance threshold of *p*‐value < 0.05. All graphs and visual representations of the data were created using GraphPad Prism software (Prism 9; GraphPad Software 2020). In contrast, as leaf gas exchange data were measured only at a single site in a single year (IL‐2023), genotype comparisons were analysed using unpaired two‐sample *t*‐tests, with statistical significance determined at *p*‐value < 0.05.

## Conflicts of Interest

The authors declare no conflicts of interest.

## Supporting information


**Figure S1:** Total fatty acid (TFA) concentration (g kg^−1^) in (a) leaf and (b) stem tissues under four environmental conditions (IL‐2023, IL‐2024, NE‐2023, and NE‐2024) grouped by genotype of TX430 background (wild‐type (WT) and oil sorghum (OS) lines (TxHO‐2and TxHO‐3)). The error bars represent the standard error. Lowercase letters indicate mean separation at *α* = 0.05 from highest to lowest value.
**Figure S2:** Total fatty acid (TFA) (g kg^−1^) in (a) leaf and (b) stem tissues under three environmental conditions (IL‐2023, IL‐2024, and NE‐2023) grouped by genotype of Ramada background (wild‐type (WT) and oil sorghum (OS) lines (RmHO‐1 and RmHO‐2)). The error bars represent the standard error. Lowercase letters indicate mean separation at *α* = 0.05 from highest to lowest value.
**Figure S3:** Fibre composition (g kg^−1^) of TX430 background (wild‐type (WT) and oil sorghum lines (TxHO‐2 and TxHO‐3)) grouped by four environmental conditions (IL‐2023, IL‐2024, NE‐2023, and NE‐2024). Measured components include: (a) cellulose, (b) hemicellulose, (c) lignin, and (d) ash concentrations. The error bars represent the standard error. Lowercase letters indicate mean separation at *α* = 0.05 from highest to lowest value.
**Figure S4:** Fibre composition (g kg^−1^) of Ramada background (wild‐type (WT) and oil sorghum lines (RmHO‐1 and RmHO‐2)) grouped by three environmental conditions (IL‐2023, IL‐2024, and NE‐2023). Measured components include: (a) cellulose, (b) hemicellulose, (c) lignin, and (d) ash concentrations. The error bars represent the standard error. Lowercase letters indicate mean separation at *α* = 0.05 from highest to lowest value.
**Figure S5:** Photographs of TX430, the abnormal growth of TxHO‐3, which appeared as a twisted whorl in IL. Photo taken on 23 August 2023.
**Figure S6:** Photographs of Ramada, lodging after a heavy windstorm event in IL on 25 August 2023. Photo taken on 27 August 2023.
**Figure S7:** Photographs of Ramada, lodging after a heavy windstorm event in NE on 31 July 2024. Photo taken on 1 August 2024.
**Table S1:** The growing degree day (GDD) of booting and harvest date in TX430 and Ramada background at the field trials in IL and NE across 2 years.
**Figure S8:** Monthly precipitation and temperature at the experimental sites in IL and NE across the 2 years of the study (2023–2024), including monthly and the 30‐year monthly average (1995–2024).
**Table S2:** Soil characteristics at the depths of 0–15 and 15–30 cm for each growing environment in IL and NE during the 2023–2024 growing seasons.
**Table S3:** Planting, booting, and harvesting dates of oil sorghum trials in IL and NE (2023–2024).

## Data Availability

Data will be made available on request.
