# Supplementary material for: Multisite Field Evaluation of Oil Accumulation and Agronomic Performance in Grain and Sweet Sorghums Engineered for Lipid Hyperaccumulation
Source: Plant Biotechnol J. 2026 Mar 25;24(7):4546–60. doi: 10.1111/pbi.70654 (PMC13278542; doi:10.1111/pbi.70654)
Supplement: Supplementary file 1 — Figure S1: Total fatty acid (TFA) concentration (g kg−1) in (a) leaf and (b) stem tissues under four environmental conditions (IL‐2023, IL‐2024, NE‐2023, and NE‐2024) grouped by genotype of TX430 background (wild‐type (WT) and oil sorghum (OS) lines (TxHO‐2and TxHO‐3)). The error bars represent the standard error. Lowercase letters indicate mean separation at α = 0.05 from highest to lowest value. Figure S2: Total fatty acid (TFA) (g kg−1) in (a) leaf and (b) stem tissues under three environmental conditions (IL‐2023, IL‐2024, and NE‐2023) grouped by genotype of Ramada background (wild‐type (WT) and oil sorghum (OS) lines (RmHO‐1 and RmHO‐2)). The error bars represent the standard error. Lowercase letters indicate mean separation at α = 0.05 from highest to lowest value. Figure S3: Fibre composition (g kg−1) of TX430 background (wild‐type (WT) and oil sorghum lines (TxHO‐2 and TxHO‐3)) grouped by four environmental conditions (IL‐2023, IL‐2024, NE‐2023, and NE‐2024). Measured components include: (a) cellulose, (b) hemicellulose, (c) lignin, and (d) ash concentrations. The error bars represent the standard error. Lowercase letters indicate mean separation at α = 0.05 from highest to lowest value. Figure S4: Fibre composition (g kg−1) of Ramada background (wild‐type (WT) and oil sorghum lines (RmHO‐1 and RmHO‐2)) grouped by three environmental conditions (IL‐2023, IL‐2024, and NE‐2023). Measured components include: (a) cellulose, (b) hemicellulose, (c) lignin, and (d) ash concentrations. The error bars represent the standard error. Lowercase letters indicate mean separation at α = 0.05 from highest to lowest value. Figure S5: Photographs of TX430, the abnormal growth of TxHO‐3, which appeared as a twisted whorl in IL. Photo taken on 23 August 2023. Figure S6: Photographs of Ramada, lodging after a heavy windstorm event in IL on 25 August 2023. Photo taken on 27 August 2023. Figure S7: Photographs of Ramada, lodging after a heavy windstorm event in NE on 31 July 2024. [file PBI-24-4546-s001.docx]

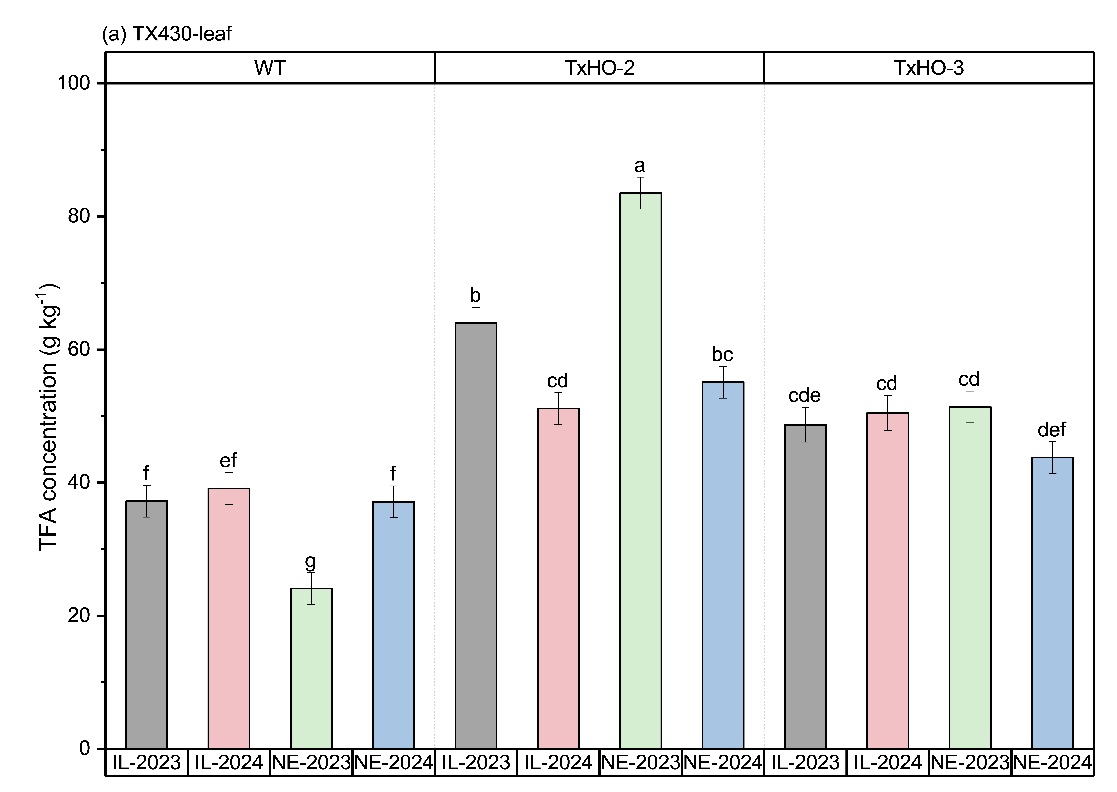

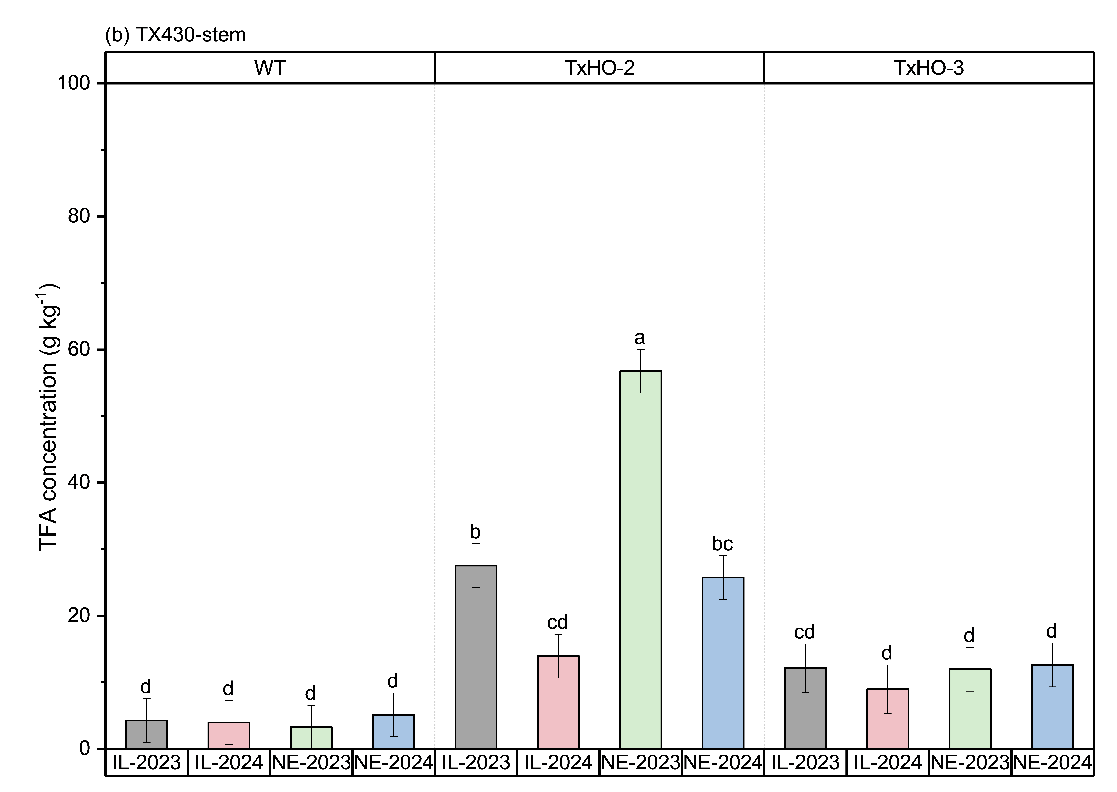


Figure S1 Total fatty acid (TFA) concentration (g kg⁻¹) in (a) leaf and (b) stem tissues under four environmental conditions (IL-2023, IL-2024, NE-2023, and NE-2024) grouped by genotype of TX430 background (wild-type (WT) and oil sorghum (OS) lines (TxHO-2and TxHO-3)). The error bars represent the standard error. Lowercase letters indicate mean separation at α = 0.05 from highest to lowest value.


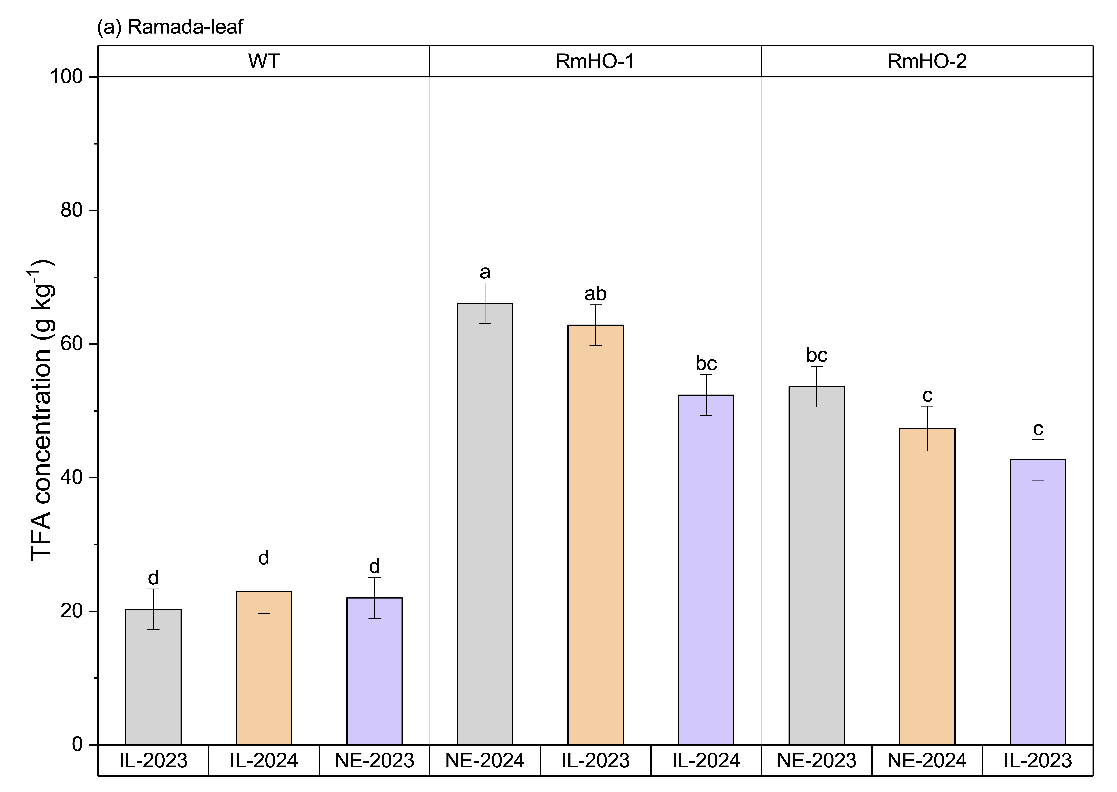

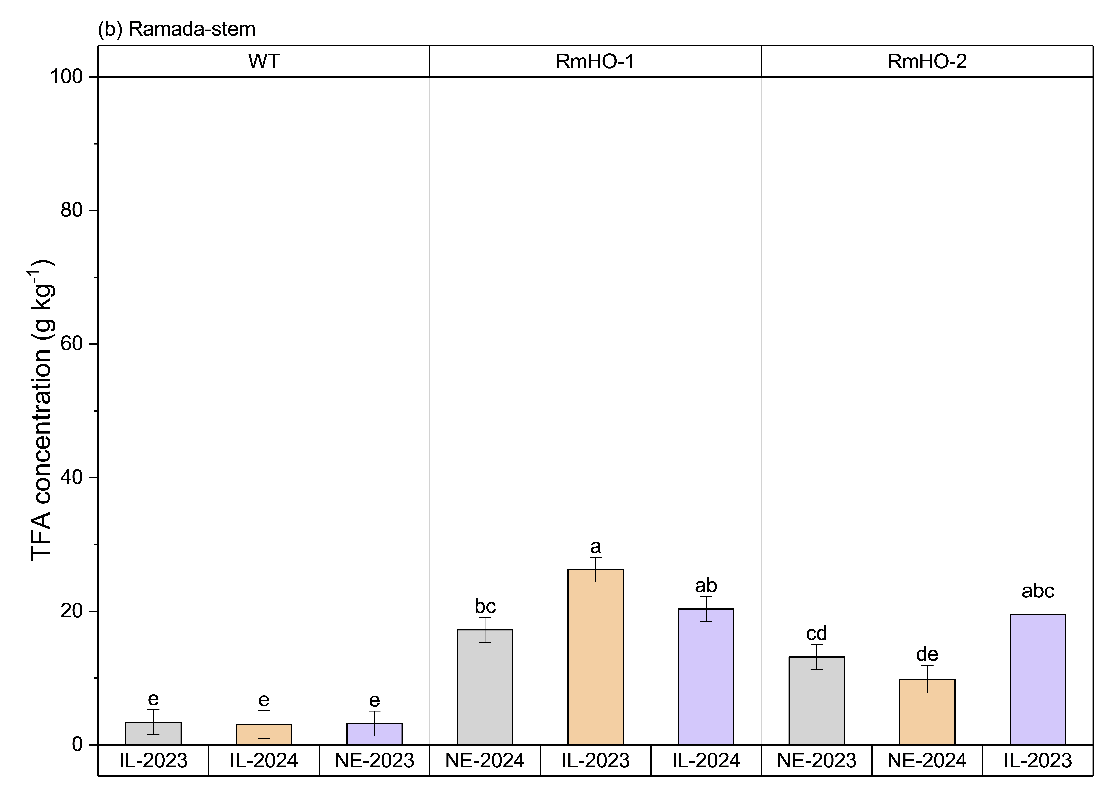


Figure S2 Total fatty acid (TFA) (g kg⁻¹) in (a) leaf and (b) stem tissues under three environmental conditions (IL-2023, IL-2024, and NE-2023) grouped by genotype of Ramada background (wild-type (WT) and oil sorghum (OS) lines (RmHO-1 and RmHO-2)). The error bars represent the standard error. Lowercase letters indicate mean separation at α = 0.05 from highest to lowest value.





Figure S3 Fiber composition (g kg⁻¹) of TX430 background (wild-type (WT) and oil sorghum lines (TxHO-2 and TxHO-3)) grouped by four environmental conditions (IL-2023, IL-2024, NE-2023, and NE-2024). Measured components include: (a) cellulose, (b) hemicellulose, (c) lignin, and (d) ash concentrations. The error bars represent the standard error. Lowercase letters indicate mean separation at α = 0.05 from highest to lowest value.





Figure S4 Fiber composition (g kg⁻¹) of Ramada background (wild-type (WT) and oil sorghum lines (RmHO-1 and RmHO-2)) grouped by three environmental conditions (IL-2023, IL-2024, and NE-2023). Measured components include: (a) cellulose, (b) hemicellulose, (c) lignin, and (d) ash concentrations. The error bars represent the standard error. Lowercase letters indicate mean separation at α = 0.05 from highest to lowest value.


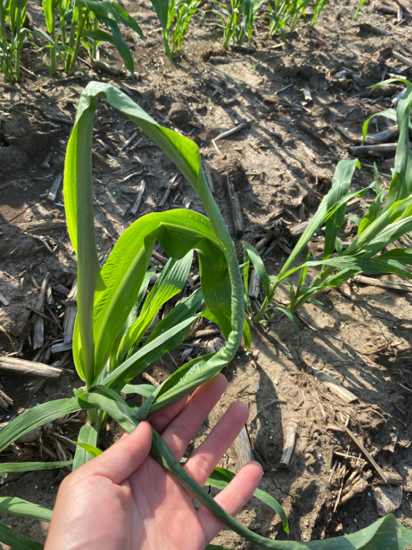


Figure S5 Photographs of TX430, the abnormal growth of TxHO-3, which appeared as a twisted whorl in IL. Photo taken on August 23, 2023


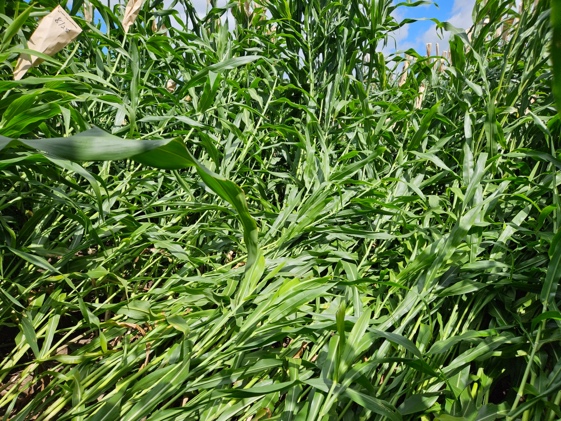


Figure S6 Photographs of Ramada, lodging after a heavy windstorm event in IL on August 25, 2023. Photo taken on August 27, 2023


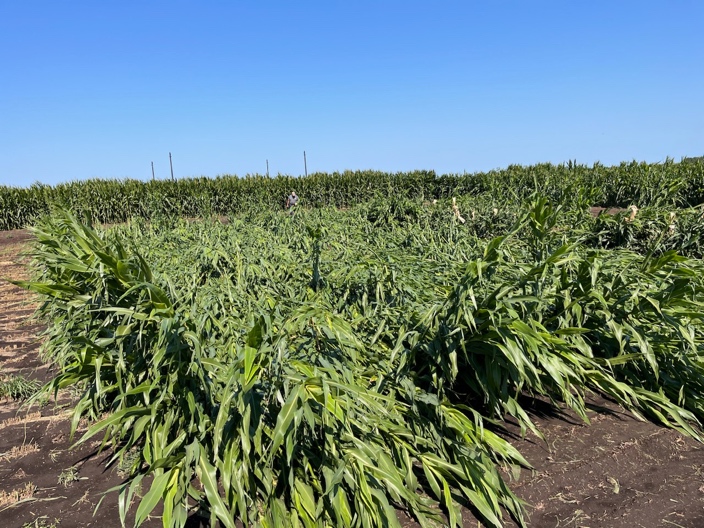


Figure S7 Photographs of Ramada, lodging after a heavy windstorm event in NE on July 31, 2024. Photo taken on August 01, 2024

Table S1 The growing degree day (GDD) of booting and harvest date in TX430 and Ramada background at the field trials in IL and NE across two years.

| Location | Background | Growth stage | Year | DAS | GDD |
| --- | --- | --- | --- | --- | --- |
| IL | TX430 | Booting | 2023 | 71 | 828 |
|  |  |  | 2024 | 70 | 822 |
|  |  | Harvest | 2023 | 110 | 1207 |
|  |  |  | 2024 | 104 | 1221 |
|  | Ramada | Booting | 2023 | 78 | 905 |
|  |  |  | 2024 | 87 | 1024 |
|  |  | Harvest | 2023 | 116 | 1257 |
|  |  |  | 2024 | 126 | 1430 |
| NE | TX430 | Booting | 2023 | 73 | 810 |
|  |  |  | 2024 | 70 | 795 |
|  |  | Harvest | 2023 | 115 | 1242 |
|  |  |  | 2024 | 117 | 1273 |
|  | Ramada | Booting | 2023 | 84 | 923 |
|  |  | Harvest | 2023 | 138 | 1437 |

Abbreviation: DAS, Day after sown


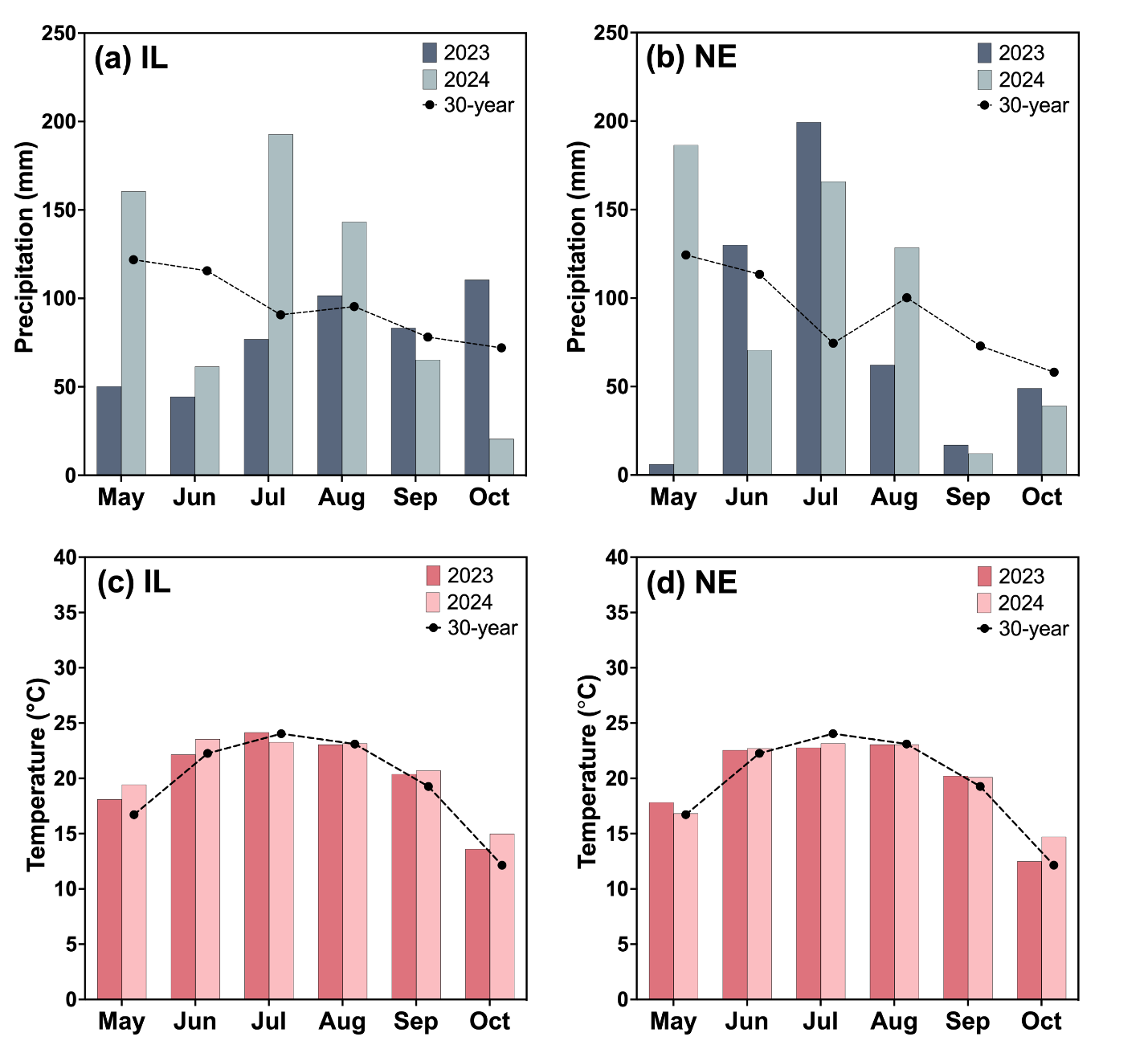


Figure S8 Monthly precipitation and temperature at the experimental sites in IL and NE across the two years of the study (2023-2024), including monthly and the 30-year monthly average (1995–2024).

Table S2 Soil characteristics at the depths of 0-15 and 15-30 cm for each growing environment in IL and NE during the 2023- 2024 growing seasons

| Location | Year | Depth (cm) | pH | Organic Matter (%) | Cation Exchange Capacity (meq 100g-1) | Phosphorus (mg/kg) | Potassium (mg/kg) |
| --- | --- | --- | --- | --- | --- | --- | --- |
| IL | 2023 | 0-15 | 6.3±0.1 | 4.1±0.0 | 14.7±0.3 | 44.2±2.6 | 208±11.8 |
|  | 2023 | 15-30 | 6.6±0.0 | 3.5±0.1 | 14.2±0.3 | 25.5±3.2 | 113.8±11.3 |
|  | 2024 | 0-15 | 6.6±0.1 | 4.4±0.1 | 14.3±0.3 | 44.9±2.8 | 172.9±7.5 |
|  | 2024 | 15-30 | 6.8±0.1 | 3.9±0.1 | 12.9±0.5 | 26.6±3.4 | 90.1±7.6 |
| NE | 2023 | 0-15 | 6.8±0.1 | 4.0±0.0 | 15.8±0.2 | 18.5±1.1 | 239±4.2 |
|  | 2024 | - | - | - | - | - | - |
| - indicates that no data were collected | | | | | | | |

Table S3 Planting, booting, and harvesting dates of oil sorghum trials in IL and NE (2023-2024).

| Location | Year | Planting date | Background | | | |
| --- | --- | --- | --- | --- | --- | --- |
|  |  |  | TX430 | Ramada | TX430 | Ramada |
|  |  |  | Booting date (DAS) | | Harvest date (DAS) | |
| IL | 2023 | June 15 | 71 | 78 | 109 | 116 |
|  | 2024 | May 23 | 70 | 87 | 104 | 126 |
| NE | 2023 | May 26 | 73 | 84 | 115 | 138 |
|  | 2024 | May 30 | 70 | - | 117 | - |
| Abbreviation: DAS, Day after sown  - indicates that no data were collected due to severe lodging caused by a windstorm in NE 2024 | | | | | | |
